# Gene–Phenotype Associations Involving Human-Residential Bifidobacteria (HRB) Reveal Significant Species- and Strain-Specificity in Carbohydrate Catabolism

**DOI:** 10.3390/microorganisms9050883

**Published:** 2021-04-21

**Authors:** Shijie Liu, Zhifeng Fang, Hongchao Wang, Qixiao Zhai, Feng Hang, Jianxin Zhao, Hao Zhang, Wenwei Lu, Wei Chen

**Affiliations:** 1 State Key Laboratory of Food Science and Technology, Jiangnan University, Wuxi 214122, China; 6180112148@stu.jiangnan.edu.cn (S.L.); zhifengf@jiangnan.edu.cn (Z.F.); hcwang@jiangnan.edu.cn (H.W.); zhaiqixiao@jiangnan.edu.cn (Q.Z.); zhaojianxin@jiangnan.edu.cn (J.Z.); zhanghao@jiangnan.edu.cn (H.Z.); 2 School of Food Science and Technology, Jiangnan University, Wuxi 214122, China; 3 (Yangzhou) Institute of Food Biotechnology, Jiangnan University, Yangzhou 225004, China; fenghang0427@outlook.com; 4 National Engineering Research Center for Functional Food, Jiangnan University, Wuxi 214122, China; 5 Beijing Innovation Centre of Food Nutrition and Human Health, Beijing Technology and Business University (BTBU), Beijing 100048, China

**Keywords:** bifidobacteria, carbohydrate, glycoside hydrolase, gene–phenotype associations

## Abstract

Bifidobacteria are among the first colonizers of the human gastrointestinal tract. Different bacterial species use different mechanisms for utilization of various carbon sources in order to establish themselves in the complex microbial ecosystem of the gut. However, these mechanisms still need to be explored. Here, a large gene–phenotype correlation analysis was carried out to explore the metabolic and genetic diversity of bifidobacterial carbohydrate utilization abilities. In this study, we used 21 different carbohydrates to determine the growth phenotypes, the distribution of glycoside hydrolases (GHs), and gene clusters related to the utilization of multiple carbon sources in six human-residential *Bifidobacterium* species. Five carbohydrates significantly stimulated growth of almost all strains, while the remaining sugars exhibited species- and strain-specificity. Correspondingly, different *Bifidobacterium* species also had specific GHs involved in fermentation of plant or host glycans. Moreover, we analyzed several carbohydrate utilization gene clusters, such as 2-fucosyllactose (2′FL), sialic acid (SA), and fructooligosaccharide (FOS). In summary, by using 217 bifidobacterial strains and a wide range of growth substrates, our research revealed inter- and intra-species differences in bifidobacterial in terms of carbohydrate utilization. The findings of this study are useful for the process of developing prebiotics for optimum growth of probiotics, especially *Bifidobacterium* species.

## 1. Introduction

Much experimental data has shown significant differences in the abundance and composition of bifidobacteria in the intestines of people of different ages [1]. Studies show that bifidobacteria represent one of the first microbes to colonize the human intestinal tract [2]. In infancy, bifidobacterial species are vertically transmitted via the mother [3]. After weaning, bifidobacterial species abundance decreases, and accounts for approximately 4.4% of the adult intestinal flora and exhibits a declining trend with age [4]. From their composition, bifidobacteria can be loosely divided into two types according to different age groups of people, “infant-type” and “adult-type”. Infant-type bifidobacteria mainly include *Bifidobacterium bifidum*, *Bifidobacterium longum* subsp. *infantis*, *Bifidobacterium breve*, and *Bifidobacterium longum* subsp. *longum* [5]. Adult-type bifidobacteria largely comprise *Bifidobacterium adolescentis*, *Bifidobacterium pseudocatenulatum*, and *Bifidobacterium longum* subsp. *longum* [6,7]. The composition of bifidobacteria is affected by many factors, among which changes in diet may be the main reason for the transition from infant-type to adult-type [8]. During breastfeeding, the large amount of indigestible human milk oligosaccharides (HMOs) ingested by infants selectively stimulates the growth of infant-type bifidobacteria [9]. After weaning, the intake of breast milk decreases, and the solid dietary components gradually increase, driving intestinal adult-type bifidobacteria colonization [10,11]. In addition, studies have found that the abundance of *B. breve* in the transitional phase of the two diets is high, but the reason for this phenomenon is unclear. As mentioned above, certain indigestible carbon sources that people consume daily have a stimulating effect on the growth of bifidobacteria in the gut. Changing dietary composition may impact health; therefore, studying the ability of bifidobacteria to utilize carbohydrates is very important, and can be used as a theoretical guide for the rational design of new “prebiotic-probiotic” products. Studies have shown that various carbon sources can selectively stimulate the growth of bifidobacteria [12]. Some diet-derived carbon sources include oligosaccharides (e.g., fructooligosaccharides (FOS), xyloligosaccharides (XOS)), polysaccharides (e.g., inulin, resistant starch, and cellulose) [13], and other host-derived carbohydrates such as HMOs and mucin [14,15,16]. Oligosaccharides recognized as “Bifidus factors” such as FOS have excellent stimulatory effects on most bifidobacteria, as had been shown in clinical trials [17,18,19]. At the same time, bifidobacteria exhibit species-specific differences in utilizing carbohydrates. For example, only a few bifidobacterial strains can grow on inulin or mucin as a single carbon source, and other strains need to grow through cross-feeding activities [20]. Nevertheless, the utilization phenotype of many carbon sources such as citrus pectin or chitooligosaccharide (COS) by some bifidobacterial species is still unknown. Therefore, experiments are needed to explore the utilization characteristics of these carbohydrates by bifidobacteria.

Since the first whole-genome sequence of *B. longum* NCC2705, part of the genetic basis of carbon source utilization of bifidobacteria can be explained from the perspective of genomics [21]. Existing genomic data show that the genome of bifidobacteria comprises approximately 12–14% of genes related to sugar utilization [22]. According to the classification of carbohydrate active enzymes (CAZy) database, the pan-genome of Bifidobacterium genus contains a large number of genes that encode glycoside hydrolases, glycosyltransferases, and carbohydrate esterases [23]. Notably, glycoside hydrolases encoded by bifidobacteria are mainly responsible for degradation of carbon sources [24]. Moreover, bifidobacterial genomes encode multiple gene clusters responsible for the utilization of various glycans, usually including one or more GHs (intracellular enzymes and extracellular enzymes), transport systems (ABC, PTS, and MFS), and transcriptional regulators [25,26]. Compared with other species, bifidobacterial genomes encode more gene clusters encoding GH13 family enzymes, such as α-amylase, pullulanase, and α-glucosidase, which can degrade starch-related carbon sources [13,27]. Different *Bifidobacterium* species may use different strategies for carbohydrate assimilation, reflecting their different adaptation abilities to host niches. However, these mechanisms are not fully understood.

Although many studies have focused on carbohydrate utilization by bifidobacteria, their ability to utilize food-derived carbon sources and their genetic basis have not been fully explained. Different bifidobacterial species may use different molecular mechanisms for utilizing the same carbon source. In the actual production process, it is necessary to understand these differences when compounding functional foods containing “prebiotic-probiotics”. Therefore, we focused on the phenotypes and genotypes of food-derived carbohydrate utilization by six bifidobacterial species (*B. longum*, *B. breve*, *B. adolescentis*, *B. infantis*, *B. pseudocatenulatum*, and *B. bifidum*) that mainly exist in the intestines of the Chinese population. The purpose of this study was to explore differences in the utilization phenotypes of different carbon sources (e.g., plant, milk-like synthetic, host-derived, and animal), and to associate possible gene clusters for revealing adaptive mechanisms of bifidobacteria.

## 2. Materials and Methods

### 2.1. Bacterial Strains and Growth Conditions

The 217 bifidobacterial strains selected in this study (Table S1) were isolated from human feces from different regions of China. All strains were sequenced and the accession numbers are also shown in Table S1. Strains were cultured in a chamber (AW500SG, Electrotek Scientific Ltd., West Yorkshire, UK) in an anaerobic atmosphere (10% (*v*/*v*) H_2_, 10% (*v*/*v*) CO_2_, and 80% (*v*/*v*) N_2_]. The bacteria were cultured in modified de Man–Rogosa–Sharpe (mMRS) medium supplemented with 0.05% (*w*/*v*) l-cysteine hydrochloride (Shanghai Macklin Biochemical Co., Ltd., Shanghai, China) (MRSC), and incubated at 37 °C for 24–48 h.

### 2.2. Carbohydrate Utilization Characterization

Twenty-one carbohydrates were selected for carbohydrate utilization experiments (Table 1). Except for 2’FL, other sugars were purchased from Shanghai Canspec Scientific Instruments Co. Ltd., Shanghai, China. According to the manufacturer, mucin is extracted from porcine gastric. The XOS is from corn cob and has a degree of polymerization between 2 and 7. The IMO is from corn starch, and consisted of glucose linked by α-1,6 glycosidic bonds. The arabinogalactan (AG) we tested is type I arabinogalactan. 

Different sugars replaced glucose in mMRS medium to a final concentration of 0.5% (*w*/*v*) as the sole carbon source for the growth of bifidobacteria, with pH adjusted to 6.8, and autoclaved at 121 °C for 15 min. All strains grew well on glucose medium and had a limit background growth on the sugar-free medium. The limit background growth was defined as a slight increase of the OD above the background on the sugar-free medium. Therefore, glucose and sugar-free medium were used as positive and negative controls, respectively. 

Bifidobacteria were grown on different carbon sources in 96-well plates. After sub-culturing, a 1% culture was inoculated into test media and cultured anaerobically at 37 °C for 48 h. For growth experiments performed in microtiter plates, a microplate reader (Varioskan Lux, Thermo, Waltham, MA, USA) was used to measure the optical absorbance at a wavelength of 600 nm (OD_600_). Experiments were carried out in triplicate, and the results are expressed as the means of these replicates. The results are shown as a heatmap, based on the following optical density cut-off values: (no growth = OD_600_ < 0.3; growth = OD_600_ > 0.3).

### 2.3. Comparative Genomics and Orthology Predictions

An all-versus-all BLASTP search (E-value 1e-3 cut-off) was performed on protein sequences of each bifidobacteria species using Orthofinder [28], as previously described [29]. The sequences were clustered into orthogroups (OGs) using the Markov cluster algorithm (MCL).

### 2.4. Glycoside Hydrolase Distribution Statistics and Signal Peptide Prediction

The carbohydrate-active enzyme gene profiles of all strains were predicted and analyzed based on similarity to the carbohydrate-active enzyme (CAZy) database (http://www.cazy.org/, accessed on 19 March 2021) by using HMMER (E-value 1e-5 cut-off) on dbCAN2 meta server (http://bcb.unl.edu/dbCAN2/, accessed on 19 March 2021). The number of GHs in each strain was counted, and the results were shown as a heatmap using TBtools [30]. The presence of signal peptides at the 5’ termini of protein sequences was analyzed using the signalP 5.0 software (http://www.cbs.dtu.dk/services/SignalP/, accessed on 19 March 2021).

### 2.5. Gene–Phenotype Correlation Analysis

Two distinct methods were used to determine correlations between phenotypes (positive or negative) and genotypes. We used IBS software to draw locus maps of gene clusters predicted to be involved in carbohydrate hydrolysis [31]. For some of the sugars commonly used by bifidobacteria, we manually searched for genes related to specific carbohydrate utilization of each species according to gene annotations, and constructed locus maps of gene clusters.

For other carbohydrates with different phenotypes, Phenolink (https://trac.nbic.nl/phenolink/, accessed on 19 March 2021) was used to perform gene–phenotype correlation analysis, similar to analyses involving different bacteria [32]. Orthologous gene group files output by Orthofinder were processed into a binary matrix (values 0 for absence and 1 for presence). The same method was used to process fermentation results to form a binary matrix of phenotype (values 0 for no growth and 1 for growth). These two files were inputs for Phenolink, together with annotation files of orthologous groups. Phenolink automatically performs analyses for all phenotypes, and we summarized these results as gene clusters. The results are shown in Table S3.

## 3. Results

### 3.1. Bifidobacteria Exhibited Differences in Carbohydrate Utilization

**Figure 1:**
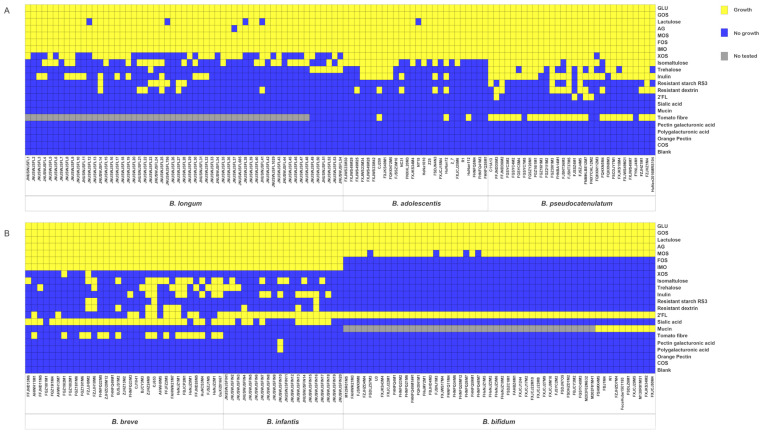
Evaluation of carbohydrate utilization. (**A**), Heatmap illustrating the growth performance of adult-type bifidobacterial strains on different carbon sources. (**B**), Heatmap showing the growth performance of infant-type bifidobacterial strains on different carbon sources.

A total of 217 strains were used for in vitro growth assays (Table S1), involving 21 carbohydrates as the sole carbon source (Table 1; Figure 1). Glucose, galactooligosaccharides (GOS), mannose oligosaccharides (MOS), arabinogalactan (AG), and lactulose supported growth of almost all strains. Except for *B. bifidum*, other species grew well on FOS and isomaltooligosaccharides (IMO). None of the strains displayed growth on citrus pectin, COS. Bifidobacteria showed species- or strain-specificities in the fermentation of some sugars tested (Table 1; Figure 1). For example, all *B. adolescentis* and *B. pseudocatenulatum* strains, nearly half of *B. longum* and only two of *B. breve* were able to grow on XOS. Similarly, almost all *B. pseudocatenulatum*, nearly half of *B. longum*, *B. adolescentis*, *B. breve*, and *B. infantis* strains could grow on isomaltulose. Interestingly, these specific phenomena were strongly reflected when bifidobacteria fermented host-derived carbohydrates. All strains of *B. infantis* and *B. bifidum*, 12 of *B. breve*, and 4 of *B. pseudocatenulatum* grew well on 2′-fucosyllactose (2’FL). However, *B. longum* and *B. adolescentis* could not utilize 2’FL. Utilization of SA was only observed in *B. breve* and *B. infantis*. Likewise, only *B. bifidum* could degrade mucin.

### 3.2. Differences in GH Distribution Characteristics of Bifidobacteria

**Figure 2:**
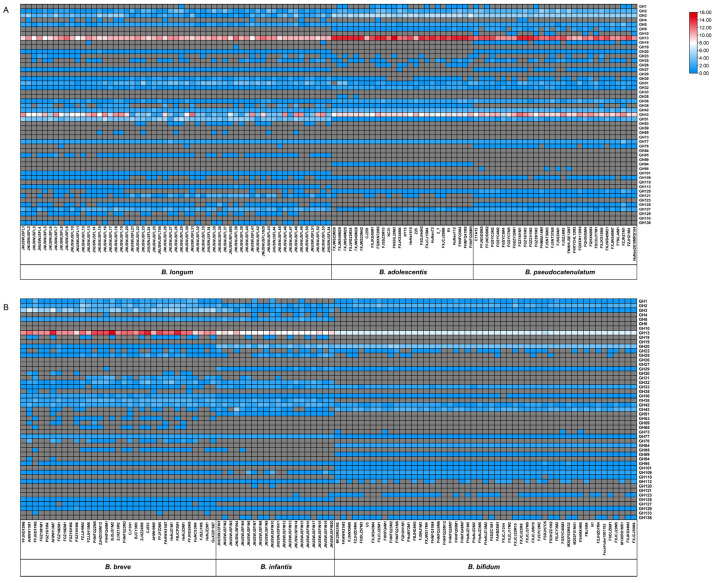
Heatmap displaying the distribution and abundance of glycoside hydrolase (GH) family genes across *Bifidobacterium*. Gene copy number of each of 48 GH families ranging from gray (absent) to red. (**A**), Heatmap illustrating the distribution and abundance of GH family genes across adult-type bifidobacteria. (**B**), Heatmap illustrating the distribution and abundance of GH family genes across infant-type bifidobacteria.

**Figure 3:**
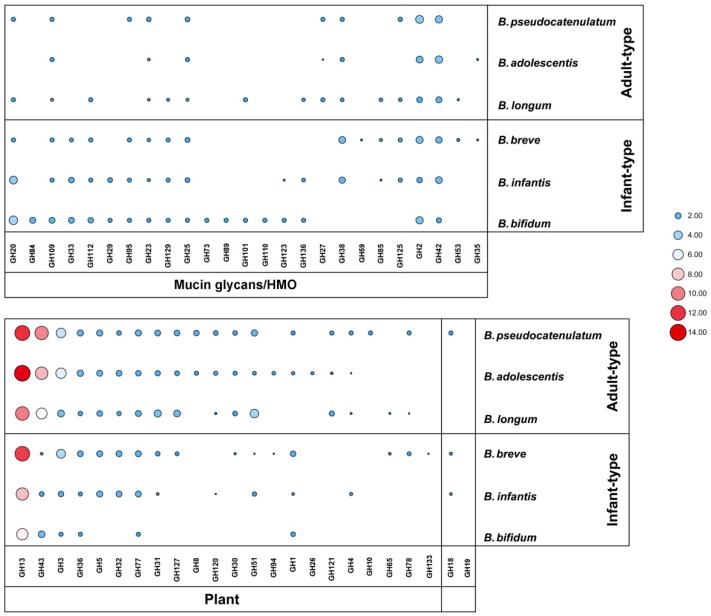
Distribution of average gene copy number of GHs in *Bifidobacterium*.

To gain insight into how the various strains showed differences in carbohydrate utilization, we counted the genomes of all strains in the experiment (Table S1). The results showed that bifidobacterial genomes encoded 49 of the 168 GH families in the CAZy database (Figure 2 and Figure 3). The annotations and primary functions of glycoside hydrolases from different GH families are presented in Table S2.

For the average number of GH genes, adult-type bifidobacteria contained more than infant-type bifidobacteria. *B. pseudocatenulatum* genome contained 58 GH genes on average, with the highest content among the six species. In contrast, *B. bifidum* genome contained 31 GH genes on average, with the lowest content among the six species. As expected, adult-type bifidobacteria encoded more GHs that responsible for catabolism of plant-related carbohydrates than infant-type bifidobacteria. In contrast, GHs related to host-derived carbohydrates degradation were more prevalent in infant-type bifidobacteria, especially in *B. bifidum* (Figure 3).

The GH13 family was the most abundant in studied bifidobacterial genomes, with an average GH copy number ranging from 7 (*B. bifidum*) to 14 (*B. adolescentis*), and which is mainly responsible for the hydrolysis of *α*-glucosidic bonds in starch-related carbohydrates. The second most frequently GHs present in adult-type bifidobacterial genomes are belonging to the GH43 family, with an average GH copy number ranging from 6 (*B. longum*) to 10 (*B. pseudocatenulatum*). However, it exhibited a lower content in infant-type bifidobacterial genomes, with an average GH copy number ranging from 1 (*B. breve*) to 3 (*B. bifidum*). Other GH families, such as GH3 and GH32, also were widely present in studied genomes. For GHs involved in degradation of host-derived carbohydrates, β-hexosaminidases (GH20) are on average represented by four paralogs in *B. bifidum* and *B. infantis* but are absent in *B. adolescentis* genomes. The sialidase (GH33) only found in infant-type bifidobacteria and is related to the degradation of mucin and sialyllactose derived from the host. Similarly, α-fucosidase (GH95) was mainly present in infant-type bifidobacterial genomes, and the genomes of 4 *B. pseudocatenulatum* strains also encoded α-fucosidase, which may target 2’FL degradation. All genomes of tested strains encoded β-galactosidase (*LacZ* of GH2, *galG* of GH42) that may also involve in degradation of milk-like synthetic carbohydrate.

### 3.3. Species-Specific GHs in Bifidobacteria

Some enzymes in specific GH families are encoded only in one species and are regarded as species-specific GHs (Figure 3). The endo-1,4-β-xylanase (GH10) only encoded in *B. pseudocatenulatum* genomes. The galactosylceramidase (GH59) and amylo-α-1,6-glucosidase (GH133) are only found in *B. breve*. The endo-β-N-acetylglucosaminidase (GH73), Nagh (GH84), AgnB (GH89), and α-1,3-galactosidase (GH110) are only present in the genome of *B. bifidum* and are related to the hydrolysis of host-derived glycans. Chitinase (GH19) only found in the genome of *B. longum* JNUSWJSFL38.

### 3.4. Secreted GHs in Bifidobacteria

**Figure 4:**
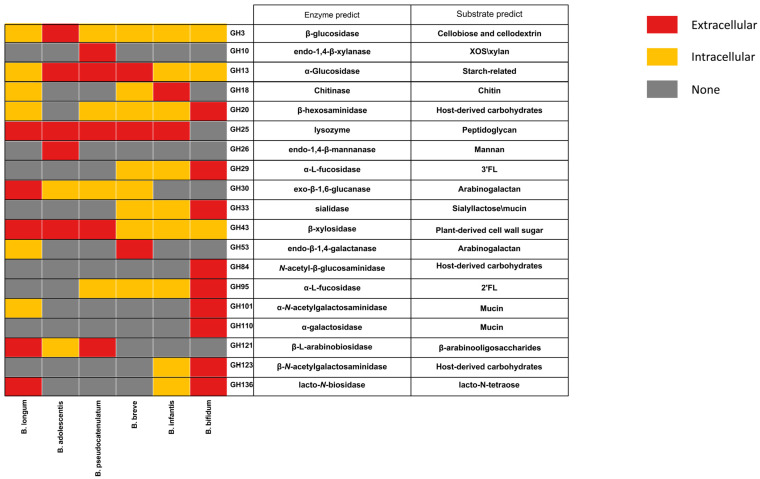
Distribution characteristics of predicted extracellular GHs across *Bifidobacterium*.

Some GHs are extracellular enzymes, giving bifidobacteria the ability to degrade complex long-chain carbohydrates, and the possibility of cross-feeding with other bacteria [14,22,33]. The predicted extracellular enzymes of bifidobacteria are distributed across 19 GH families, mainly in GH13, GH43, and GH25 families (Figure 4). Compared to other species, *B. bifidum* encodes more extracellular GHs involved in degradation of host-derived glycans, such as GH29 (α-fucosidase), GH33 (sialidase), GH84 (N-acetyl β-glucosaminidases), and GH95 (α-fucosidase). It is worth noting that certain GHs are predicted to have different cellular localization in different *Bifidobacterium* species or strains. For example, β-xylosidase (GH43_4) is a predicted intracellular enzyme in some *B. longum* strains, but a predicted extracellular enzyme in *B. pseudocatenulatum*. GH33 and GH95 families enzymes were predicted intracellular enzymes in *B. infantis* and *B. breve*, while they were predicted extracellular enzymes in *B. bifidum*. The endo-β-galactanase (GH53) only found in *B. breve* and *B. longum*, was a predicted extracellular enzyme in all *B. breve* strains. However, only three *B. longum* strains (*B. longum* JNUSWJS1029, *B. longum* JNUSWJS685, and *B. longum* JNUSWJSFL26) were predicted to encode the extracellular endo-β-galactanase.

### 3.5. 2’FL Utilization Gene Clusters Analysis

**Figure 5:**
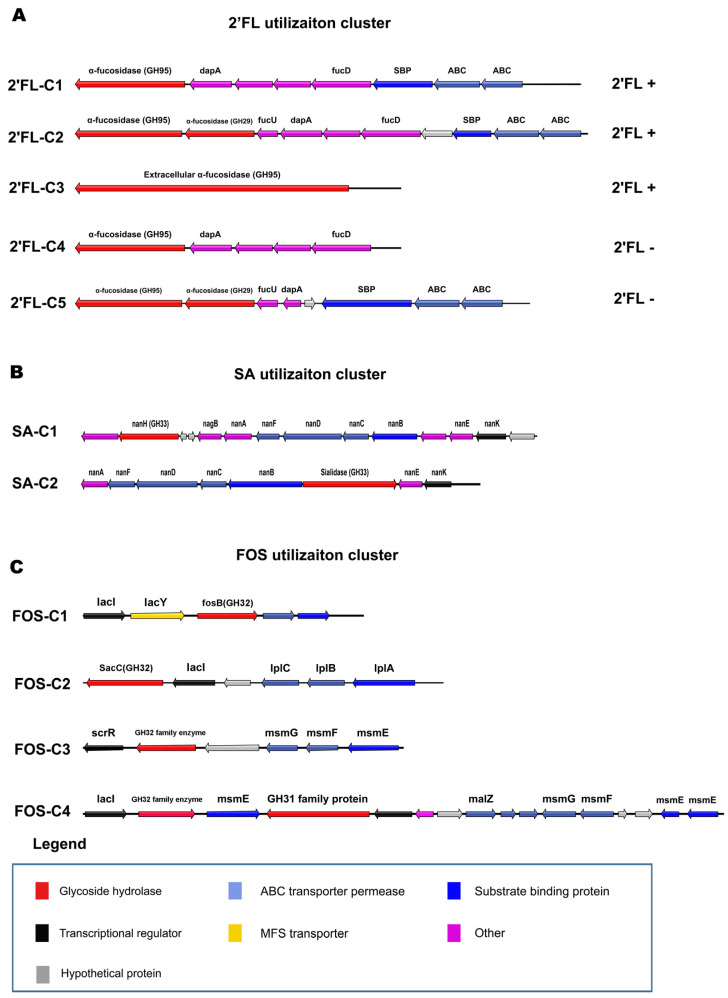
Locus map representing predicted carbohydrate utilization clusters. (**A**) 2’FL utilization clusters. (**B**) SA utilization clusters. (**C**) FOS utilization clusters.

According to our genotype–phenotype association analysis, there were three types of gene clusters related to 2’FL utilization in bifidobacteria (Figure 5A, Table S3). Based on the localization of the enzymes, 2’FL-C1 is an intracellular degradation gene cluster of *B. breve* and *B. pseudocatenulatum* with growth phenotype of 2′FL. 2’FL-C2 also represents an intracellular degradation gene cluster in all *B. infantis* strains, while 2’FL-C3 represents an extracellular degradation gene cluster of *B. bifidum*. In addition, 2’FL-C4/C5 are gene clusters in *B. breve* strains which cannot utilize 2’FL.

2’FL-C1 is the basic gene cluster for 2’FL degradation in 12 *B. breve* and 4 *B. pseudocatenulatum* strains, including α-fucosidase of GH95, 4 putative fucose degradation genes, and the ABC transport system. Compared with 2’FL-C1, 2’FL-C2 contains an additional *α*-fucosidase of GH29 and putative fucose isomerase *FucU*. The *α*-fucosidase of GH29 may be related to the degradation of 3’FL, and may endow *B. infantis* with more extensive HMOs assimilation abilities. In addition, the remaining 21 *B. breve* strains encoded 2’FL-C4/C5 that lack ABC transporters or *fucD* (fuconate dehydratase) cannot utilize 2’FL, even though genomes of these strains encode α-fucosidases (GH95 or GH29). Since the α-fucosidases (GH95) of *B. bifidum* are extracellular enzymes, they can degrade 2′FL extracellularly. However, due to the lack of fucose catabolism genes, *B. bifidum* can only import lactose for growth [34].

### 3.6. Sialic Acid Utilization Gene Cluster Analysis

Previous studies have identified that the metabolism of sialic acid by *B. breve* is mediated by a coordinated action of *N*-acetylneuraminate lyase (NanA), *N*-acetylmannosamine kinase (NanK), and N-acetylmannosamine-6-phosphate 2-epimerase (NanE) [35]. Based on the homology analysis, two types of gene clusters are involved in the hydrolysis of SA in bifidobacteria (Figure 5B; Table S3), all of which contain sialidases of GH33. The gene cluster SA-C1 was significantly associated with the utilization of SA by *B. breve*, including a sialidase, multiple predicted sialic acid degradation genes (*nanK*, *nanE,* and *nanA*), ABC transporters, and a transcriptional regulator. SA-C2 is found in *B. infantis*, and the structure is similar to SA-C1. Due to lack of SA-C1/C2, some *B. breve* and *B. infantis* strains could not grow in SA. Although *B. bifidum* has two extracellular sialidases, the genomes of all studied *B. bifidum* strains lack corresponding SA hydrolysis genes so that this species could not use SA as a single carbon source for growth.

### 3.7. FOS and Inulin Utilization Gene Cluster Analysis

The result showed that strains encoding β-fructofuranosidase (GH32) had FOS utilization ability. In contrast, all tested *B. bifidum* strains lack GH32 family enzyme and could not grow on FOS. We found that there were four types of gene clusters related to FOS degradation (Figure 5C; Table S3). FOS-C1 is encoded in the genomes of all strains of *B. longum*, *B. breve*, *B. adolescentis*, *B. pseudocatenulatum*, and *B. infantis*, except for *B. bifidum.* Strains containing two copies of GH32 family genes encode both FOS-C1 and FOS-C2 clusters. FOS-C3/C4 are specifically encoded in the genomes of *B. breve*.

FOS-C1 is composed of *lacI*, β-fructofuranosidase (GH32), MFS transport system *lacY*, and the ABC transport system, which may confer bifidobacteria the basic ability to utilize FOS or sucrose. Compared with FOS-C1, FOS-C2 does not contain MFS transporters, which are predicted to transport different structures of FOS or inulin, giving certain strains increased fructan utilization abilities. Few *B. breve* strains contain 3–4 copies of GH32 family enzymes, which are encoded in FOS-C3/C4. The structure of FOS-C3 is similar to that of FOS-C2 but contains different types of regulators and transporters. FOS-C4 has *β*-fructosidase and α-glucosidase, as well as their corresponding transporters and regulatory systems. Gene-trait matching (GTM) analysis found that FOS-C4 is related to the inulin utilization of *B. breve*. The six *B. breve* strains whose genomes contained FOS-C4 all could degrade inulin for growth. In addition, inulin hydrolysis may also be implicated in substrate specificity of *β*-fructosidase encoded by GH32 enzymes in different strains.

### 3.8. XOS Utilization Gene Cluster Analysis

**Figure 6:**
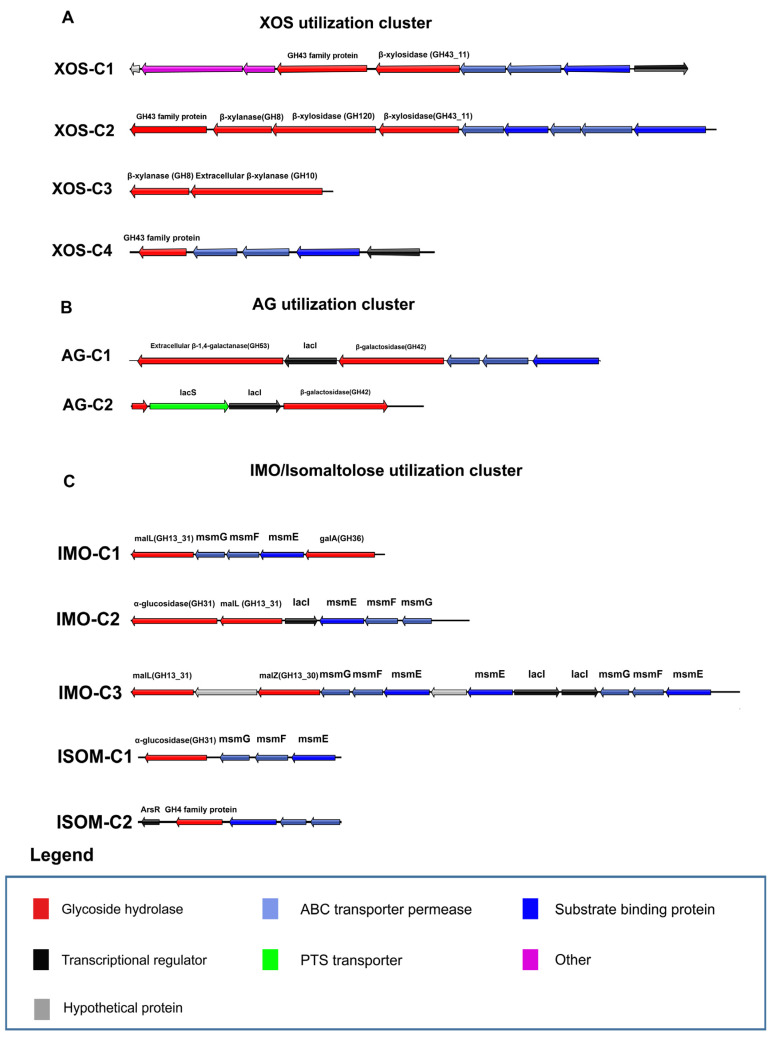
Locus map representing predicted carbohydrate utilization clusters. (**A**) XOS utilization clusters. (**B**) AG utilization clusters. (**C**) IMO/isomaltulose utilization clusters.

Previous studies have identified several GH43 family enzymes in bifidobacteria that degrade xylan-related carbon sources [36]. In addition, the enzymes belonging to GH8, GH10, and GH120 families may also participate in XOS or xylan degradation [37,38]. Through our association analysis, there are four types of gene clusters related to the utilization of XOS in bifidobacteria (Figure 6A; Table S3). XOS-C1 is encoded in the genomes of *B. adolescentis*, *B. pseudocatenulatum,* and *B. longum* strains with growth on XOS. Notably, 26 *B. longum* strains encode XOS-C1 showed growth on XOS, while remaining 31 *B*. *longum* strains lack of this cluster with no growth. Therefore, XOS-C1 is a key gene cluster for *B. longum* to utilize XOS. It is consisted of two GH43 family enzymes (β-xylosidase from GH43_11 and α-L-arabinofuranosidase from GH43_12), several predicted xylose degradation genes, and the corresponding ABC transport system. Addtionally, XOS-C2 is encoded in the genome of all *B. adolescentis* and *B. pseudocatenulatum* strains. It compose of α-L-arabinofuranosidase (G43_10), other GHs related to xylan degradation from GH8, GH120 and GH43_11 families, and the ABC transport system. Specifically, XOS-C3 is encoded in the genomes of nine *B. pseudocatenulatum* strains and consisted of two β-xylanases (GH8 and GH10). Moreover, β-xylanase (GH10) is a predicted extracellular enzyme, which may confer *B. pseudocatenulatum* the ability to degrade long-chain xylans extracellularly. XOS-C4 is encoded in 13 *B. breve* strains, but only 2 strains could use XOS for growth. It is consisted of a transcriptional regulator, ABC transport system and a GH43 family enzyme.

### 3.9. Type I AG Utilization Gene Cluster Analysis

Type I AG consists of a chain of β-1,4-linked D-galactopyranose linkages, and previous studies have mentioned that type I AG can be degraded by the combination of β-1,4-galactanase and β-1,4-galactosidase [39]. Except for the GOS/lactulose degradation gene clusters (Figure S1), we also found that 17 strains of *B. breve* and 19 strains of *B. longum* encode β-1,4-galactanase of GH53, which is predicted to participate in the degradation of type I AG (Table S3). Seventeen *B. breve* and 3 *B. longum* strains encode AG-C1, which is composed of extracellular β-1,4-galactanase of GH53, β-galactosidase of GH42, and the ABC transport system. The AG-utilizing gene cluster of the remaining 16 *B. longum* strains is AG-C2. Different from AG-C1, it contains a PTS transport system instead of the ABC transporters and a gene with the loss of GH53 domain.

### 3.10. IMO and Isomaltulose Utilization Gene Cluster Analysis

The α-glucosidases from GH13_31, GH31, and GH4 families are related to hydrolyzed IMO and isomaltulose. Since the composition of monosaccharides is different, we assumed that there might be different pathways for these sugars. Except for *B. bifidum*, α-glucosidase (GH13_31) is present in all genomes of the other five species. Moreover, strains that did not encode enzymes belonging to GH31 and GH4 families could also utilize IMO, so the α-glucosidase (GH13_31) is predicted to be essential for IMO degradation.

There were three types of gene clusters containing α-glucosidases of GH13_31 (Figure 6C, Table S3). IMO-C1 is found in the genomes of 5 bifidobacterial species (*B. longum*, *B. breve*, *B. adolescentis*, *B. pseudocatenulatum*, and *B. infantis*) and is predicted to be the basic gene cluster for the hydrolysis of maltose-related substrates. Five *B. adolescentis* and 10 *B. pseudocatenulatum* strains encoded IMO-C2. IMO-C3 is present in 37 strains of *B. longum* and 5 strains of *B. breve*. From the perspective of structural characteristics, IMO-C1 contains *α*-glucosidase, *α*-galactosidase, and the ABC transport system. Compared with IMO-C1, IMO-C2 contains one more *α*-glucosidase of GH31 and *lacI*. IMO-C3 consists of two α-glucosidase and ABC transporter. These two gene clusters may confer bifidobacteria the ability to hydrolyze the glucoside bonds of different structures. The influences of OD value by presence of IMO-C2/C3 and the function of IMO-C2/C3 still need to be characterized by further experiments.

Since isomaltulose utilization phenotype of bifidobacteria was strain-specific, IMO-C1 may not be main cluster for isomaltulose utilization. GTM analysis revealed that isomaltulose-C1 is related to the hydrolysis of isomaltulose by *B. breve*, and isomaltulose-C2 is related to the hydrolysis of isomaltulose by *B. infantis* (Figure 6C; Table S3). Isomaltulose-C1 is composed of α-glucosidase (GH31) and an ABC transport system. Isomaltulose-C2 is composed of the *ArsR* transcriptional regulator, maltose-6-phosphate glucosidase (GH4), and the ABC transport system. However, the presence of isomaltulose-C1 does not fully prove that bifidobacteria can hydrolyze isomaltulose. This is because isomaltulose-C1 is present in all *B. longum*, *B. adolescentis*, and *B. pseudocatenulatum* strains, but their utilization of isomaltulose is still strain-specific.

## 4. Discussion

The ability of bifidobacteria to metabolize plant-related or host-derived carbohydrates is essential for their persistence in the intestinal tract [40]. This work reveal the knowledge about species-specific and strain-specific variation in carbohydrate utilization within the bifidobacteria isolated from people of various ages and different regions of China. Moreover, from the perspective of species, *B. pseudocatenulatum* had the broadest utilization abilities for the carbon sources in this experiment. *B. adolescentis* and *B. longum* preferred plant-related carbon sources, but some strains grew weakly. Unlike other species, *B. breve* and *B. infantis* can utilize host-derived SA. Compared with the other bifidobacteria species, *B. bifidum* had a weaker ability to utilize plant-related carbohydrates and could only utilize MOS and AG. In contrast, it showed good utilization of host-derived carbon sources and was the only species able to utilize mucin. According to our results, *B. breve* displayed more obvious strain-specificity than other species when fermenting plant- and host-derived carbohydrates, whereas *B. bifidum* exhibited the opposite trend. Previous reports have also pointed out that *B. breve* has a wide and variable carbon source utilization capacity [41]. However, because *B. breve* lacks specific GH43 family enzymes involved in xylan and arabinoxylan degradation, this species is more adapted to the infant gut environment. *B. bifidum* mainly exists in the infant intestinal tract and has a specific host-derived carbon source utilization capacity [42].

The carbohydrate active enzymes encoded by bifidobacteria control the metabolism of carbohydrates, and are the basis for adapting to different niches [43]. Corresponding to the results of phenotypic experiments, adult-type bifidobacteria encoded a large number of GHs from GH8, GH13, GH43, and GH51 families involved in the metabolism of complex plant glycans. In contrast, infant-type bifidobacteria encoded more GHs from GH20, GH29, GH33, and GH95 families related to degradation of host-derived glycans. This result was similar to that previously described for GHs distribution in bifidobacteria, indicating that the evolution of bifidobacteria species may be guided by the selective adaptation of different carbon sources [12]. Species-specific GHs also reflect the unique ecological and environmental adaptative mechanisms of different *Bifidobacterium* species, and may promote interactions with other microorganisms. Notably, strains belonging to the same species exhibited different patterns of GH gene representation (Figure 2), which may be one of the key factors in designing suitable “probiotic–prebiotic” products.

Previous studies have identified several fucosidases and FL transporters for the hydrolysis of 2’FL in *B. infantis*, *B. bifidum* and *B. kashiwanohense* strains, and these genes play a crucial role in the degradation of fucosylated HMOs with different structures [44,45,46,47,48,49]. Through our data, we also found that the structure of these gene clusters differed between strains and the phenotype characteristics of each species. For example, *B. breve* strains which lack suitable transporters or *fucD* cannot grow on 2’FL. Extracellular hydrolysis of 2’FL by *B. bifidum* may provide cross-feeding with other bifidobacteria [34]. A study has also reported that *B. longum* SC596 encodes a gene cluster similar to 2’FL-C2 in *B. infantis* [50], but we could not identify any homologous gene clusters or fucosidases in selected *B. longum* strains. Previous studies also found that *B. infantis* mainly contains two types FL transporters (FL1-BP and FL2-BP) with distinct but overlapping specificities, both of which could affect the utilization abilities of 2′FL [51]. Generally, studies indicate that utilization of 2’FL is characteristic of bifidobacteria isolated from the intestinal tract of infants [45,51]. However, in our experiments, about 42% (5/12) *B. breve* and 100% (4/4) *B. pseudocatenulatum* strains isolated from adult feces grew well on 2’FL. A previous study has also found that the FL transporter may bind to fucosylated substrates other than fucosylactose [48]. Therefore, we speculate that the existence of 2’FL utilization gene clusters in *B. breve* or *B. pseudocatenulatum* may be strain-specific and not fully related to host age. More strains and related analysis are needed to confirm these correlations.

Similar to previous reports, gene clusters containing alpha-sialidases (GH33) also encode a transporter and hydrolase genes for SA [35]. In the actual intestinal environment, *B. breve* often requires other species such as *B. bifidum* to degrade mucin or sialyllactose extracellularly in order to obtain SA. For example, co-cultured with mucin as a single carbon source drives cross-feeding interaction between *B. bifidum* PRL2010 and *B. breve* UCC2003 [20].

The universal utilization of lactulose and GOS shows the importance of β-1,4-galactosidase for bifidobacteria. The gene clusters encoding β-galactosidases also encode various transporters (ABC, MFS, and PTS), and several GOS ABC transporters have been characterized in *B. breve* [52,53,54]. When *B. breve* UCC2003 grows on lactulose, transcription of *LacZ*2 (Bbr_0285) and *LacZ*6 (Bbr_1552) are up-regulated by 39.7 and 35.9 times, respectively [55]. However, the substrate specificities of other transporters still require experimental validation (Figure S1A). Surprisingly, our data also show the widespread growth of bifidobacteria on Type I AG, which is different from previous studies [39,56]. We speculated that a small part of Type I AG we tested may hydrolyzed due to sterilization, which would support the growth of almost all strains. Previous studies have demonstrated that multiple β-galactosidases and β-galactanases are required for Type I AG degradation. The extracellular enzyme *GalA* (GH53) in AG-C1 is similar to the reported sequence of *B. longum* NCC2705, *B. longum* DJ010, and *B. breve* UCC2003 [56], and can degrade Type I galactan and Type I AG into disaccharides/trisaccharides. In contrast, certain *B. longum* strains appear to undergo an internal deletion with the loss of the special gene domain, so they cannot utilize galactan [39].

The experiment results showed that most HRB species had related gene clusters to degrade FOS, except for *B. bifidum*. Previous studies have identified that the β-fructofuranosidases from GH32 family is the key to FOS degradation [57], similar to our results. This study also found multiple FOS utilization gene clusters in bifidobacteria. FOS-C1 and FOS-C2 have been characterized in several strains [58,59]. FOS-C3/C4 seems to represent species-specific clusters of *B. breve* genes, and its function needs to be characterized. The ability of bifidobacteria to degrade different fructan chain lengths is strain-specific [60]. This may be due to the substrate specificity of β-fructofuranosidases and transporters encoded by different strains [61,62]. Therefore, compared with the strains containing only one copy, the strains containing multiple copies of the GH32 family enzymes may have the ability to utilize fructans of multiple structures. The GH32 family enzymes encoded by the bifidobacteria in our experiment were predicted to be intracellular, so it may not degrade inulin carbohydrates with a high degree of polymerization. Consistent with previous studies, we observed that many strains grow poorly on inulin [17]. Some strains with growth phenomena may hydrolyze short-chain structures.

This study showed that *B. adolescentis* and *B. pseudocatenulatum* had strong XOS utilization abilities, while others were strain-specific. Several enzymes related to XOS or arabinoxylooligosaccharides (AXOS) degradation have been characterized in bifidobacterial species, and they have different substrates specificities [37,63,64,65]. For example, two β-xylosidase, XylB from GH120 family and XylC from GH43 family, with different specificities have been in vitro characterized in *B. adolescentis* [64]. Recent study has also characterized three xylosidases from GH43 family in *B. pseudocatenulatum* and these enzymes exhibit functionally identical activity in XOS degradation [36]. Moreover, extracellular β-xylanase (GH10) may provide some *B. pseudocatenulatum* strains stronger long-chain xylan utilization abilities. Therefore, in the adult intestinal environment, *B. pseudocatenulatum* may avoid excessive interspecies competition with other species such as *B. longum* or *B. breve* that use XOS with specific chain lengths, even with the possibility of cross-feeding.

The growth of 20 strains of *B. breve* in isomaltulose, as reported in previous studies, is strain-specific, which is consistent with the results of our experiments [66]. Previous reports have shown that α-glucosidase (GH13_31) can degrade IMO and isomaltulose [67]. According to the previous study, the α-glucosidase gene (GH13_31) located in IMO-C3 cluster is shown to contribute to the increased growth ability of certain *B. longum* strains on isomaltulose [68]. We also identified ISOM-C1 of *B. breve* and ISOM-C2 of *B. infantis* to be associated with isomaltulose utilization. However, it is difficult to analyze the genetic basis of isomaltulose utilization by *B. adolescentis*, and *B. pseudocatenulatum* from the perspective of genes existence. Therefore, we speculate that isomaltulose metabolism may involve multiple pathways or expression regulation, and more methods are needed for identification.

Similarly, GTM analysis also revealed that bifidobacteria contain multiple trehalose-utilizing pathways, which may be related to transporters (Figure S1B). It has been reported that Ag1 and Agl2 (GH13) in *B. breve* UCC2003 exhibit trehalose hydrolysis activity [69]. However, this strain cannot use trehalose as a sole carbon source for growth, which may be due to the lack of a corresponding transport system. GH65 (kojP, EC 2.4.1.230) in Tre-C5 may be related to trehalose utilization, but due to the lack of the PTS transport system in bifidobacteria, trehalose may not be hydrolyzed through this pathway. Similarly, in the study of *B. longum* 105-A, the authors also characterized that the α-glucosidase encoded by BL105A_1883 has the activity of degrading trehalose [68]. However, the molecular basis for strains-specific phenomenon of bifidobacteria in trehalose utilization still need to be explored. In addition, bifidobacteria in insect intestines generally have complete trehalose degradation pathways. Transcriptome data showed that when *B. coryneforme* LMG18911 utilizes trehalose, the expression of BCOR_0130 (GH13), BCOR_0130 (GT20), and related ABC and MFS transport systems in the genome is upregulated [70].

Interestingly, MOS significantly stimulated the growth of bifidobacteria in our experiments. According to the manufacturer, the MOS product is a mixture containing α- and β-type of MOS. Therefore, bifidobacteria encode at least one enzyme to degrade these two types of MOS. This research showed that different bifidobacteria species had multiple gene clusters enabling MOS degradation (Figure S1C). Mannosidase from GH2, GH5, GH26, GH38, and GH125 families may exert hydrolytic activity on MOS. Moreover, mannosidases from GH38 and GH125 families are predicted α-mannosidases. In addition, the GH5 family enzymes found in bifidobacteria are homologs of BlMan5_8, which has characterized an extracellular endo-β-(1-4)-mannanase (GH5_8) [63]. In previous studies, the specific GH26 family enzyme of MOS-C4 in *B. adolescentis* was characterized as an extracellular endo-β-(1-4)-mannanase (BaMan26A) that act on ivory nut mannan and konjac glucomannan, which consist of β-mannose residues [71]. However, no related genes encoding α- and β-mannosidase have been identified in *B. bifidum*. This shows that additional types of gene clusters degrade MOS, and it will be necessary to explore related utilization mechanisms through gene expression in the future.

In this study, the very large number of strains that were examined and the wide range of potential growth substrates that were screened. Moreover, we explored phenotype and genotype diversity related to the metabolism of several sugars by bifidobacteria. It is helpful to understand the mechanism by which bifidobacteria adapt to the human intestine and would contribute to future cooperative genomic studies. In the future, more in-depth research is needed to test and expand our current understanding.

## Figures and Tables

**Table 1:** Utilization of carbohydrates by bifidobacteria.

Origin	Carbohydrate	*B. longum*	*B. breve*	*B. adolescentis*	*B. infantis*	*B. pseudocatenulatum*	*B. bifidum*
**Plant**	**FOS**	all	all	all	all	all	none
	**IMO**	all	all	all	all	all	none
	**MOS**	all	all	all	all	all	48/52
	**AG**	56/57	all	all	all	all	all
	**XOS**	26/57	2/33	all	none	all	none
	**Isomaltulose**	25/57	14/33	15/26	6/20	28/30	none
	**Trehalose**	7/57	12/33	6/26	3/20	20/30	none
	**Resistant Starch RS3**	7/57	3/33	none	1/20	6/30	none
	**Resistant Dextrin**	6/57	8/33	5/26	3/20	12/30	none
	**Inulin**	13/57	8/33	7/26	8/20	25/30	none
	**Pectin galacturonic acid**	none	none	none	1/20	none	none
	**Polygalacturonic acid**	none	none	none	1/20	none	none
	**Orange pectin**	none	none	none	none	none	none
	**Tomato fibre**	none	17/33	3/26	none	26/30	none
**Synthesis**	**GOS**	all	all	all	all	all	all
	**Lactulose**	53/57	all	24/26	all	all	all
**Host**	**SA**	none	30/33	none	11/20	none	none
	**2’FL**	none	12/33	none	all	4/30	all
	**Mucin**	none	none	none	none	none	all
**Animal**	**COS**	none	none	none	none	none	none
	**Glucose**	all	all	all	all	all	all

## Data Availability

The accession numbers of all strains were reported in Table S1.

## Supplementary Materials

The following are available online at https://www.mdpi.com/article/10.3390/microorganisms9050883/s1, Figure S1: Locus map representing the predicted carbohydrate utilization clusters; Table S1: Strains information; Table S2: GHs distribution and function. Table S3: Genotype–phenotype association.
