# Supplementary material for: Gene–Phenotype Associations Involving Human-Residential Bifidobacteria (HRB) Reveal Significant Species- and Strain-Specificity in Carbohydrate Catabolism
Source: Microorganisms. 2021 Apr 21;9(5):883. doi: 10.3390/microorganisms9050883 (PMC8143103; doi:10.3390/microorganisms9050883)
Supplement: Supplementary file 1 [file microorganisms-09-00883-s001.zip › SUP M/Fig S1.pdf]

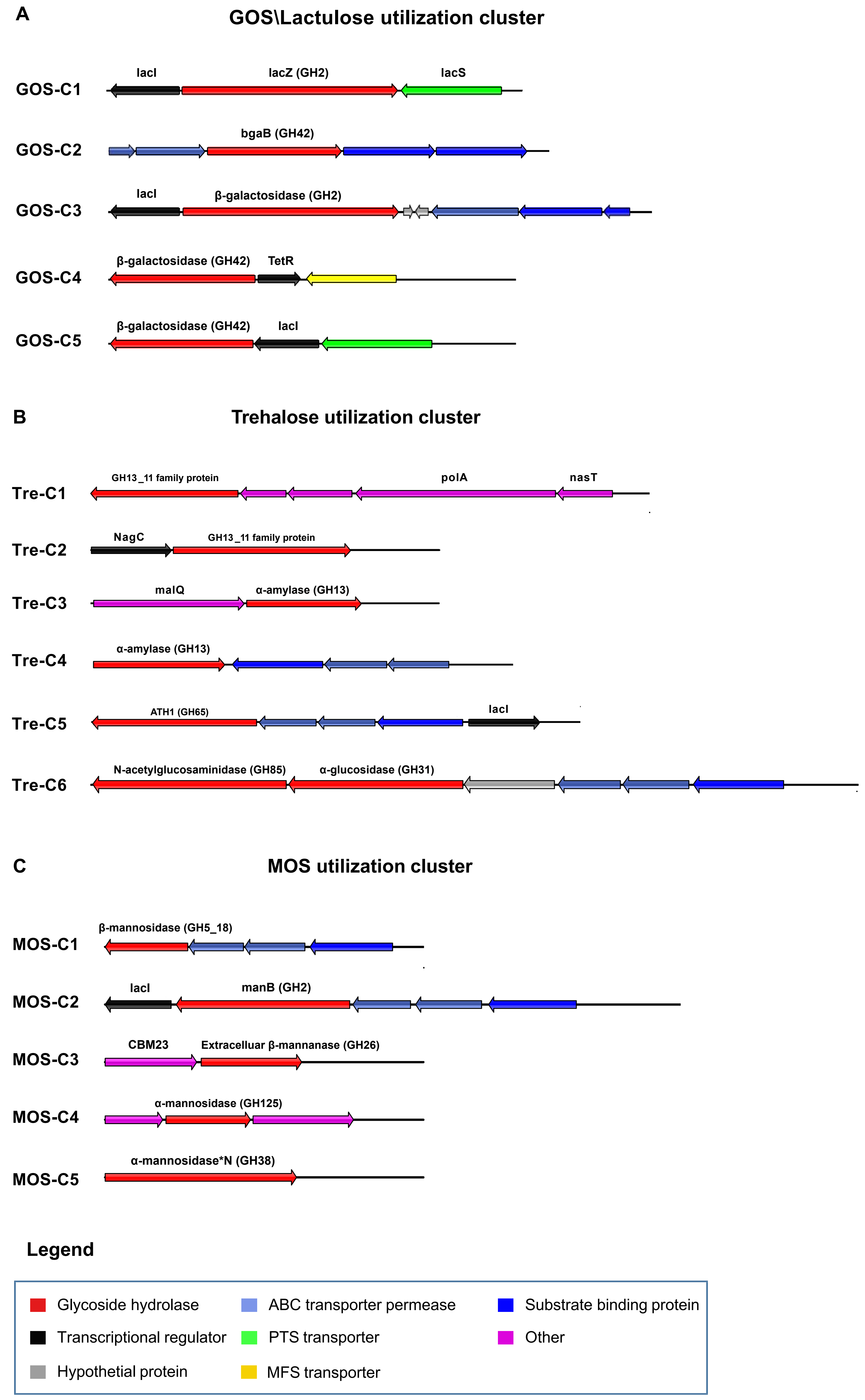

Figure S1. Locus map representing the predicted carbohydrate utilization clusters. A, GOS\ Lactuloseutilization clusters. B, Trehalose utilization clusters. C, MOS utilization clusters.
